# Impact of Ivabradine on Inflammatory Markers in Chronic Heart Failure

**DOI:** 10.1155/2016/6949320

**Published:** 2016-10-16

**Authors:** Ilonka Rohm, Daniel Kretzschmar, Rudin Pistulli, Marcus Franz, P. Christian Schulze, Christian Stumpf, Atilla Yilmaz

**Affiliations:** ^1^Department of Internal Medicine I, Friedrich-Schiller-University of Jena, Jena, Germany; ^2^Department of Internal Medicine II, Friedrich-Alexander University of Erlangen-Nuremberg, Erlangen, Germany; ^3^Department of Internal Medicine II, Elisabeth Klinikum Schmalkalden, Schmalkalden, Germany

## Abstract

*Background*. Inflammation plays a crucial role in the progression of chronic heart failure (CHF). Ivabradine is known to reduce the morbidity and mortality of patients with CHF under certain conditions. Beyond the reduction of heart rate, only limited knowledge exists about potential anti-inflammatory effects of ivabradine that might contribute to its benefit in CHF. Thus, the present study aimed to investigate the effect of ivabradine on systemic inflammation.* Methods*. In the present study, 33 patients with CHF due to dilated, ischemic, and hypertensive cardiomyopathy were treated with ivabradine according to the guidelines of the European Society of Cardiology (ESC). A number of circulating dendritic cells as well as inflammatory mediators were investigated using FACS analysis and ELISA, respectively, before and during ivabradine therapy.* Results*. Treatment with ivabradine resulted in a significant improvement of CHF symptoms as well as an increase in left ventricular ejection fraction. Moreover, ivabradine treatment led to a significant reduction of TNF-alpha (TNF-*α*) serum levels and a reconstitution of circulating dendritic cells which are known to be reduced in patients with CHF.* Conclusion*. We show that treatment with ivabradine in patients with CHF resulted in an improvement of HF symptoms and ejection fraction as well as a normalization of inflammatory mediators.

## 1. Introduction

Chronic heart failure (CHF) has a high prevalence and results in increased morbidity and mortality in the western countries [1]. Due to its enormous costs resulting from treatment with expensive drugs and devices, heart failure is a major health issue that is worth focusing on [1].

CHF is characterized by the interaction of diverse inflammatory mediators which are considered to contribute to disease progression [2]. For example, serum levels of inflammatory cytokines including interleukins- (IL-) 1, 6, and 18, TNF-*α*, and C-reactive protein are increased in CHF [3, 4]. These mediators exert deleterious effects like endothelial dysfunction and apoptosis of myocardial cells [5]. Furthermore, elevated levels of certain proinflammatory mediators, such as C-reactive protein and IL-6, correlate with disease severity and are associated with increased mortality in CHF patients [3, 4].

According to current guidelines [6], the basis of medical therapy in CHF includes ACE-inhibitors and beta blockers. A mineralocorticoid receptor (MR) antagonist is added to the medication if patients still suffer from CHF symptoms. Ivabradine is indicated for patients who remain symptomatic (NYHA classes II–IV), with a left ventricular ejection fraction ≤35%, with sinus rhythm and a heart rate ≥70/min., according to the ESC guidelines.

Ivabradine is a selective inhibitor of the hyperpolarisation activated cyclic-nucleotide-gated funny current I(f) present in cardiac pacemaker cells of the sinoatrial node [7]. This inhibition results in a reduction of resting and exercise heart rate without any known effect on cardiac contractility and blood pressure [8]. Heart rate reduction results in improved myocardial oxygen supply via a prolonged diastolic filling time, as well as improved ventricular filling. In patients with CHF, ivabradine has a beneficial effect on left ventricular remodeling [9] and survival [10].

Research investigating the mechanisms leading to the beneficial effects of ivabradine treatment showed that amongst the other effects, it prevents the alteration of endothelial cells. Luong et al. were able to demonstrate in a mouse model that ivabradine treatment suppresses the expression of proinflammatory vascular cell adhesion protein-1 (VCAM-1) and enhances the induction of anti-inflammatory endothelial nitric oxide synthase (eNOS) in the aorta. Luong et al. concluded from their experiments that ivabradine protects arteries by influencing wall shear stress and thus changing local mechanical conditions to trigger an anti-inflammatory response [11]. Li et al. also supported the thesis that ivabradine prevents low shear stress and by this reduces endothelial inflammation. They demonstrated that treatment with ivabradine reduces the generation of reactive oxygen species via the mTOR/eNOS pathway [12]. Custodis et al. showed in apolipoprotein E-deficient mice that ivabradine reduces oxidative stress and improves endothelial function [13]. Besides these studies focusing on ivabradine's effects on wall shear stress and endothelial cells, further experiments including different animal models were recently designed to investigate further anti-inflammatory effects of this drug. A special focus was set on the recently raised idea that ivabradine might exert its effect not only via heart rate reduction, but also via pleiotropic effects similar to statins. To elucidate the mechanism by which ivabradine reduces atherosclerotic plaque formation in apolipoprotein E-deficient mice, Walcher et al. aimed to investigate whether this medication regulates chemokine-induced migration of lymphocytes. In this study, they found that ivabradine reduces the chemokine-induced CD4-positive lymphocyte migration by reduction of f-actin formation and ICAM3 translocation to the uropod of the cell [14]. Furthermore, Heusch et al. [15] used a pig model and abolished the heart rate reducing effect of ivabradine using atrial pacing. They showed that, independent of heart rate reduction, ivabradine was capable of reducing the infarct size. This finding was supported by Kleinbongard et al. who observed in a mouse model that ivabradine reduces infarct size in the absence of heart rate reduction [16]. Additionally, ivabradine was demonstrated to inhibit the production of proinflammatory cytokines such as IL-6 and TNF-alpha, as well as the chemokine MCP-1 [9]. In myocarditis, treatment with ivabradine led to inhibition of myocardial fibrosis, prevention of LV-EF worsening, and improvement of survival [9].

In humans, very limited knowledge exists about the effects of ivabradine on inflammatory mediators so far and the present study aimed to further elucidate this topic. Additionally, to the best of our knowledge, no study has investigated the effect of ivabradine on professional antigen-presenting cells such as dendritic cells yet. Dendritic cells are crucial in adaptive, T cell-derived immune responses. Recent studies have given evidence that the number of circulating myeloid dendritic cells is reduced in patients with stable CHF due to ischemic and nonischemic genesis [17], but also in decompensated CHF [18]. In these studies, the reduction in circulating dendritic cells correlated with the clinical stage of CHF. This raised the question whether treatment with ivabradine might affect immunological changes, in particular the number of circulating dendritic cells as well as the serum levels of inflammatory mediators in CHF.

## 2. Material and Methods

### 2.1. Study Cohort

The patients were recruited in the present study in the outpatient clinic for heart failure of the Department of Cardiology of the University Hospital Jena, Germany. Consecutively, 50 patients were recruited into our study if they had stable CHF for more than one year and were under best medical treatment.

The recruitment was performed by a physician of the outpatient clinic for heart failure who is specialist in internal medicine as well as cardiology and whose subspecialisation is the treatment of heart failure patients. The inclusion criteria were (except for HR and LV-EF) in concordance with those of the SHIFT study [10]:

- Age > 18 years
- Sinus rhythm
- HR > 75 bpm
- LV systolic dysfunction (LV-EF < 50%)
- Ischemic, dilated, or hypertensive stable CHF > 12 months
- Standard heart failure therapy with beta blockers, ACE-inhibitors, and aldosterone-receptor antagonists (NYHA III-IV) in an adequate dosage

Exclusion criteria were those which might interfere with the analysis in our study:

- Recent cardiac decompensation (<3 months)
- Recent acute coronary syndrome (<3 months)
- Acute or chronic infections
- Malignancies
- Autoimmune diseases, for example, lupus erythematosus and rheumatoid arthritis
- Hyperthyroidism
- Medication with immunosuppressive agents

At baseline study visit, the patients were subdivided into three groups according to the etiology of CHF. The decision about the subdivision was made by the same physician who recruited the patients into the study. Patients which could not be subdivided into a subgroup since the etiology of chronic heart failure could not be clearly determined were not included in our study.

1. Ischemic cardiomyopathy (ICM; CAD proven by coronary angiography)
2. Dilated cardiomyopathy (DCM; angiographically excluded CAD, occasionally proven by biopsy, no history of hypertension)
3. Hypertensive cardiomyopathy (HCM; angiographically excluded CAD, LV Hypertrophy, and history of hypertension).

Consecutively, 12 patients were subdivided in the ICM group, 11 in the DCM group, and 10 in the HCM group.

The study protocol was approved by the local ethics committee and conforms to the principles outlined in the “Declaration of Helsinki” (1964). All patients gave informed consent.

### 2.2. Treatment and Follow-Up of the Study Participants

**Figure 1:**
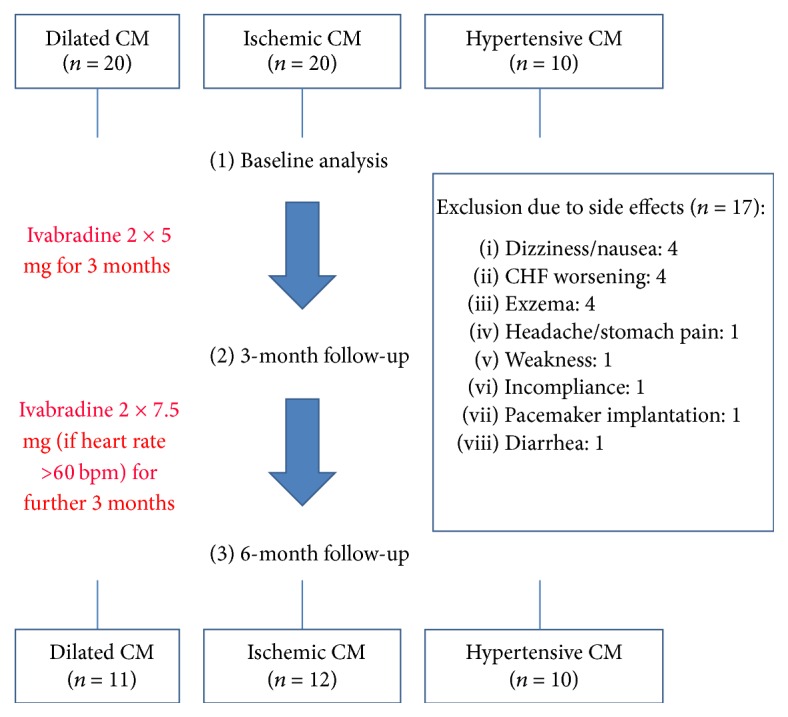
Study visits and study medication of the enrolled participants. 50 patients were included in the study and in the initial study visit; ivabradine was started on a dosage of 2 × 5 mg. In the 3-month follow-up, dosage was adjusted to 2 × 7.5 mg if heart rate was still above 60 bpm. The study ended with the 6-month follow-up. 17 patients were excluded due to side effects, so 33 patients completed the study. bpm = beats per minute, CHF = chronic heart failure, and CM = cardiomyopathy.

Figure 1 shows the chronology of treatment of the patients recruited into our study. At the baseline visit, ivabradine medication with 2 × 5 mg per day was started. Follow-up visits were performed after 3 and 6 months. If the heart rate was still above 60 bpm at the 3-month follow-up, ivabradine dosage was elevated up to 2 × 7.5 mg per day. If the heart rate was below 60 bpm, the 2 × 5 mg per day dosage was continued. Of the enrolled 50 patients, 17 had to be withdrawn from the study due to side effects mentioned in Figure 1. Phosphenes were reported but did not cause any drop-outs from the study. In 21 of the remaining study patients, the dosage was elevated up to 15 mg/d after 3 months, whereas 12 patients remained on the initial dosage of 10 mg/d.

During each study visit (baseline, 3-month follow-up, and 6-month follow-up), the following analyses were performed:

1. Evaluation of heart failure symptoms and physical examination
2. SF-36 questionnaire (The SF-36 questionnaire is a patient-reported survey of patients health including 36 items that originates from the Medical Outcome Study [19, 20]. In the present study, the German version was used, http://www.familienmedizin-bremen.de/news/SF36_ LQ_Fragebogen_01.pdf.)
3. Determination of heart failure parameters, for example, brain natriuretic peptide (BNP)
4. Echocardiography with analysis of ejection fraction (Simpson) and LVEDd (M-mode)
5. Serological analysis of inflammatory parameters: TNF-alpha, hsCRP, and IL-6 using ELISA
6. Circulating myeloid and plasmacytoid DCs using flow cytometry

### 2.3. Statistical Analysis

The statistical analysis used in this study was performed with SigmaPlot Software Version 12.0 (Systat Software Inc.). Data were tested for normal distribution using the Shapiro-Wilk test. All values are presented as mean ± standard error of the mean. *P* < 0.05 was considered statistically significant. Parameters which were normally distributed were compared with the paired Student's *t*-test, otherwise by signed rank test.

## 3. Results

### 3.1. Baseline Parameters

Table 1 shows the clinical data of the 33 study patients. Overall, the clinical data did not significantly differ between the study groups except for the occurrence of hypertension, which was expectedly higher in the group with hypertensive cardiomyopathy.

During the present study, the effects of ivabradine were investigated in two steps: (1) effects of ivabradine in all patients irrespective of the etiology of heart failure; (2) effects of ivabradine on CHF with respect to its etiology in subgroup analyses.

### 3.2. Effect of Ivabradine Therapy on the Quality of Life in CHF Determined by the SF-36 Questionnaire Irrespective of the Etiology of Heart Failure

The SF-36 questionnaire provided us information about general health and daily activities reflecting the quality of life during ivabradine therapy compared to baseline visit. The questionnaire showed us significant improvements in general health and daily activities after 6 months of ivabradine treatment. In Table 2, the significant effects of ivabradine treatment are highlighted.

### 3.3. Effect of Ivabradine Therapy on Vital, Serological and Echocardiographic Parameters in CHF Irrespective of the Etiology of Heart Failure

**Figure 2:**
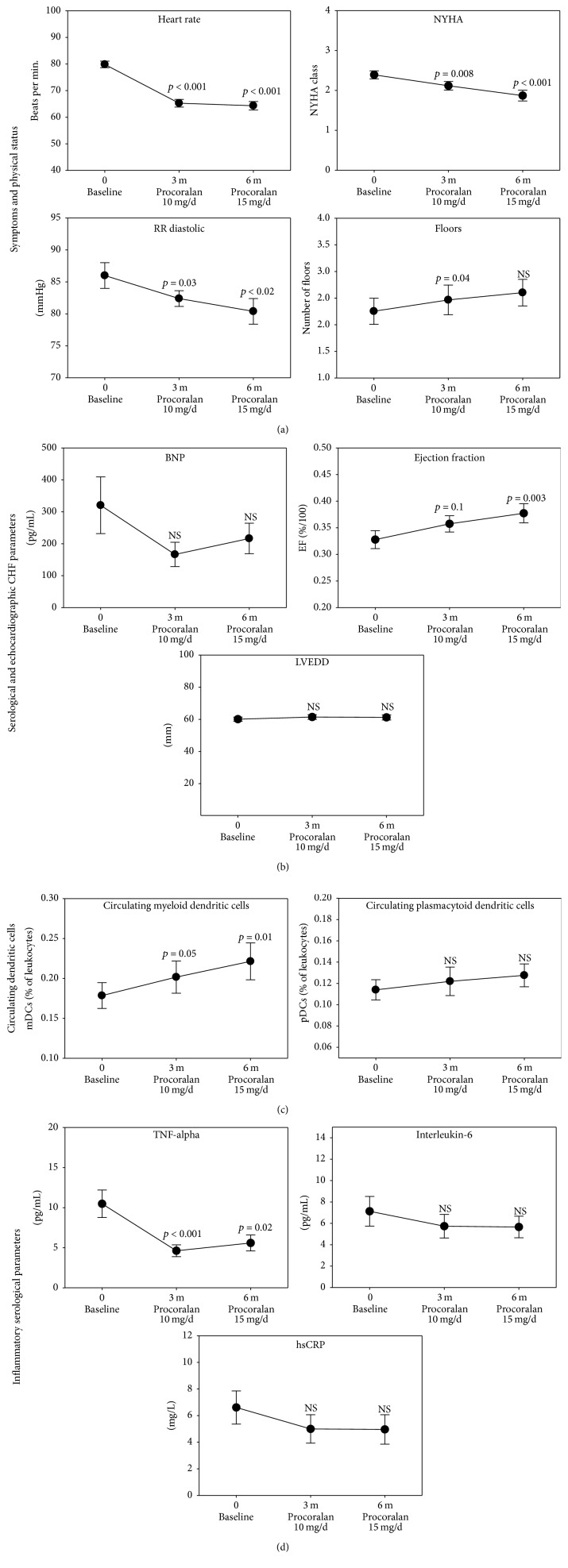
Effect of ivabradine treatment on clinical, laboratory, echocardiographic, and immunological parameters in chronic heart failure (CHF) after 3 months (10 mg/d ivabradine) and 6 months (10–15 mg/d ivabradine). (a) Vital parameters and symptoms for CHF, (b) serological and echocardiographic parameters, (c) circulating myeloid and plasmacytoid dendritic cells, and (d) inflammatory serological parameters in patients with CHF according to their ejection fraction. DCs = dendritic cells, hsCRP = high sensitive C-reactive protein, IL = interleukin, LVEDd = left ventricular end-diastolic diameter, NYHA = New York Heart Association functional class, RR sys/dia = systolic/diastolic blood pressure, and TNF-alpha = tumor necrosis factor alpha. NS = not significant.

As expected, ivabradine led to a significant reduction of the heart rate from initially appr. 80 to 64 bpm after 3 months. There was however only a slight difference between the 3 and 6 months (Figure 2(a)). It should be taken into account though that the dosage of ivabradine was elevated in only 21 of 33 patients at up to 15 mg/d in the 3-month follow-up. Additionally, ivabradine caused a significant reduction in the mean diastolic blood pressure (mean: baseline, 86 mmHg; 3 months, 82 mmHg; 6 months, 80 mmHg). Regarding CHF symptoms, a slight but significant improvement in NYHA class was observed (mean: baseline, 2.4; 3 months, 2.1; 6 months, 1.8). Additionally, the participants reported a significant improvement in their physical capacity evaluated in their ability to climb stairs (“floors”) (mean: baseline, 2.2 floors; 3 months, 2.4 floors; 6 months, 2.6 floors) (Figure 2(a)). However, no significant changes could be observed for BNP (Figure 2(b)). On the contrary, echocardiographic analysis demonstrated a statistically significant increase in the ejection fraction (mean: baseline, 33%; 3 months, 36%; 6 months, 38%). On the other hand, the LVEDd did not show any significant changes (Figure 2(b)).

Via FACS analysis, a significant increase of circulating myeloid DCs was observed after ivabradine treatment (Figure 2(c)) (mean: baseline, 0.18%; 3 months, 0.2%; 6 months, 0.22%). Plasmacytoid DCs did not show significant changes, as expected [15].

Serological analyses of proinflammatory cytokines revealed a significant reduction of TNF-*α* during ivabradine therapy (mean: baseline, 10.5 pg/mL; 3 months, 4.6 pg/mL; 6 months, 5.6 pg/mL). Levels of hsCRP and IL-6 showed only a marginal but no significant reduction during ivabradine treatment (Figure 2(d)).

### 3.4. Effect of Ivabradine on CHF Regarding the Etiology of Heart Failure

**Figure 3:**
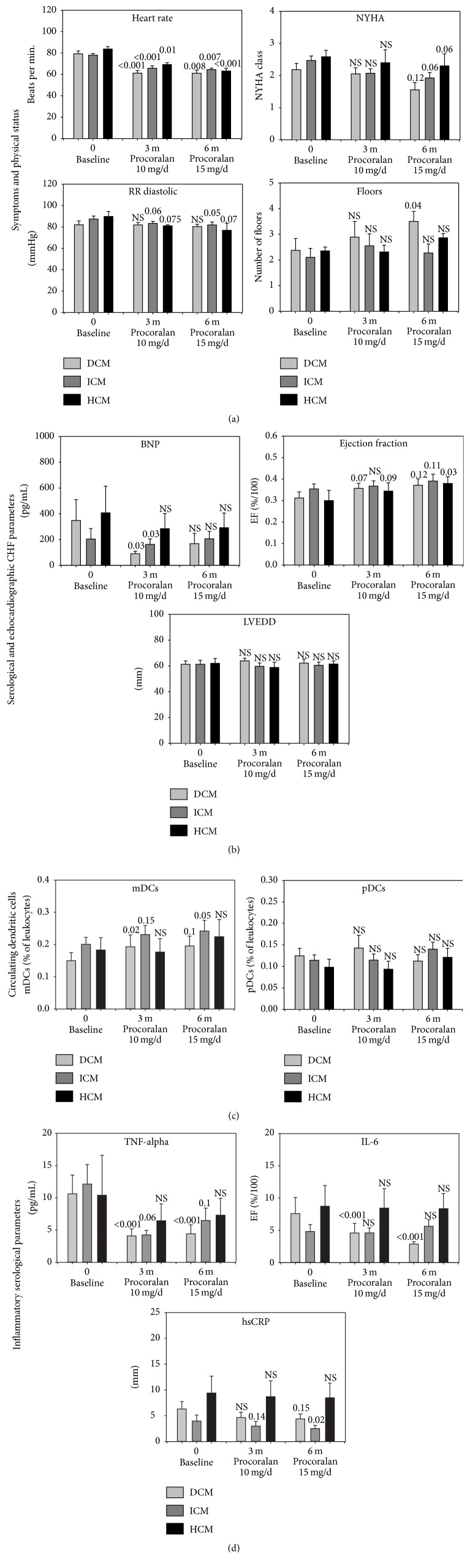
Effect of ivabradine treatment on chronic heart failure (CHF) according to its etiology after 3 months (10 mg/d ivabradine) and 6 months (10–15 mg/d ivabradine). (a) Vital parameters and symptoms for CHF, (b) serological and echocardiographic parameters, (c) circulating myeloid and plasmacytoid dendritic cells, and (d) inflammatory serological parameters in patients with CHF according to their ejection fraction. DCM = dilated cardiomyopathy, DCs = dendritic cells, HCM = hypertensive cardiomyopathy, hsCRP = high sensitive C-reactive protein, ICM = ischemic cardiomyopathy, IL = interleukin, LVEDd = left ventricular end-diastolic diameter, NYHA = New York Heart Association functional class, RR sys/dia = systolic/diastolic blood pressure, and TNF-alpha = tumor necrosis factor alpha. NS = not significant.

Subgroup analyses were performed to evaluate the effect of ivabradine dependent on the etiology of heart failure (Figure 3). The described heart rate reduction through ivabradine was observed for all subgroups. The changes in diastolic blood pressure caused by ivabradine were more pronounced in the HCM group, as expected (Figure 3). The NYHA class also improved for all subgroups, but the number of patients was not enough to reach statistical significance. The improvement of the patients' ability to climb stairs was most evident in the DCM group (Figure 3(a)). The serological analysis of BNP did not reveal any significant changes in the HCM group. For the ICM and DCM group, the decrease in serological BNP values only reached significance in the 3-month follow-up, but not in the 6-month follow-up measurements (Figure 3(b)). Echocardiographic evaluation of ejection fraction revealed a significant improvement during ivabradine therapy for HCM patients and borderline significance for the other groups (Figure 3(b)). LVEDd did not differ for any group during ivabradine treatment. FACS analysis of DCs showed a significant increase in circulating myeloid DCs especially for DCM and ICM. The HCM subgroup did not show a significant increase. For plasmacytoid DCs, no subgroup showed any effects on the number of circulating cells during ivabradine therapy (Figure 3(c)). ELISAs determining proinflammatory cytokines in participants' serum revealed different observations for the subgroups. For TNF-alpha, we detected a significant decrease in the DCM group during ivabradine therapy and an almost significant decrease in the ICM group. IL-6 was significantly reduced during ivabradine medication only in the DCM group. In contrast, in the ICM group we observed a significant reduction of hsCRP. Interestingly, no significant immunological alterations were detected in the HCM group at all during ivabradine treatment (Figure 3(d)).

## 4. Discussion

Ivabradine is known to lead to a significant reduction in morbidity and mortality of CHF patients [10]. It has recently been a point of discussion whether the benefit of ivabradine in CHF is only the result of heart rate reduction or might be explained by additional, pleiotropic effects. Inflammation is known to contribute to heart failure progression, and dendritic cells are involved in this process [18]. We recently showed a decreased number of dendritic cells in biopsy samples of DCM patients which predicts a poor prognosis. We were also able to demonstrate a decrease in circulating myeloid DCs in patients with acute myocardial infarction and their recruitment into the infarcted myocardium [21, 22]. Additionally, we and others showed that the number of circulating mDCs is reduced in patients with heart failure [17, 18]. These findings brought up the idea of the present study to investigate the effect of ivabradine on of the levels of circulating DCs and proinflammatory serum cytokines. To the best of our knowledge, this study is the first one addressing this issue in humans. Patients with CHF were recruited according to the indication for ivabradine medication as recommended in the ESC guidelines [6]. In the course of one year, 33 patients completed 6 months of ivabradine therapy, receiving 10 mg/d for 3 months and 15 mg/d for the further 3 months if the heart rate was still >60* *bmp. The present study demonstrated a significant decrease in diastolic blood pressure in patients with HCM. Interestingly, this differs from results of former studies that did not observe an effect of ivabradine on blood pressure [23]. The reason for this discrepancy is not clear, but a diastolic dip of blood pressure might be the result of the prolonged diastole with increased preload. However, echocardiographic analyses revealed no increase of aortic regurgitation as a reason for the decrease of diastolic blood pressure. In the present study, BNP showed a decrease under ivabradine treatment compared to baseline values for all subgroups. This observation is in concordance with other studies [10]. Nevertheless, for all subgroups, a U-shaped curve of BNP values was observed. In the 3-month follow-up (ivabradine 10 mg/d), a decrease in BNP was visible. In the ICM and the DCM, but not the HCM group, this decrease even reached statistical significance. In contrast, in the 6-month follow-up (ivabradine 15 mg/d), such observation was reversed: BNP reincreased in all subgroups. This finding corresponds to the results of the recently published SIGNIFY trial, in which a higher dosage of ivabradine was associated with an adverse outcome [24]. This result might suggest that a lower ivabradine dosage of 10 mg/d might be more beneficial in the treatment of CHF than the full dosage of 15 mg/d. Additionally, a significant increase in ejection fraction determined by echocardiography was observed during ivabradine therapy. Such is in concordance with a former Japanese study involving 126 patients, which showed that an ivabradine therapy with relatively low dosages of 2 × 2.5 mg or 2 × 5 mg led to a significant improvement of ejection fraction compared to the placebo group after 6 weeks of treatment [25]. Moreover, a significant increase in circulating mDCs up to values which were shown to be normal in healthy individuals [22] was observed in our study under CHF treatment with ivabradine. In the subgroup analysis, this effect was only significant for the ICM and the DCM group. In a recent study, we showed that low levels of circulating mDCs are a negative prognostic factor for CHF [17]. Thus, it can be assumed that a reconstitution in circulating myeloid DCs might be beneficial regarding the progression of CHF. For pDCs, no significant changes were observed during ivabradine treatment. However, no association between pDCs and CHF has been described [17]. We further investigated different proinflammatory cytokines, such as TNF-alpha, IL-6, and hsCRP, which are associated with the pathogenesis of CHF [26]. Ivabradine treatment caused a significant decrease of the elevated levels of TNF-alpha and a marginal reduction of IL-6 and hsCRP. For both cellular and serological immunological parameters, subgroup analysis revealed that changes during ivabradine therapy were mainly visible in patients with DCM and ICM rather than HCM. This result is in line with the inflammatory concept of the pathogenesis of these diseases. Inflammation is the main pathomechanism in the development of ICM and DCM: ICM results from coronary artery disease caused by atherosclerosis. Nowadays, atherosclerosis is considered as a chronic inflammatory process of the arterial wall. Beyond different inflammatory cells like macrophages, dendritic cells, and different subgroups of T cells, cytokines support the inflammatory response within the atherosclerotic lesion. Proinflammatory cytokines, for example, IL-6, TNF-alpha, and C-reactive protein, were found to correlate with plaque progression during atherogenesis as well as ICM, predicting major cardiovascular events [27, 28]. In the pathogenesis of DCM, inflammation also plays a pivotal role. There is accumulating evidence that myocarditis and DCM are closely related. This reveals the underlying inflammatory component in the pathogenesis of DCM. Regarding the cytokines investigated in the present study, C-reactive protein levels are related to CHF severity in DCM [29]. For TNF-alpha, a contribution to myocardial fibrosis in DCM has been suggested in the literature [30]. Inflammation has not been reported in any similar extent in connection to HCM. This might explain why the effects of ivabradine are much more pronounced in DCM and ICM, rather than in HCM. As Högye et al. also compared circulating levels of IL-6 and TNF-alpha in patients with DCM to patients with HCM, they found similar results to the ones in the present study for TNF-alpha. Whereas TNF-alpha was increased in the DCM group compared to healthy individuals, no elevated levels were found in the HCM group. However, they observed significantly elevated levels of circulating IL-6 for both the DCM and HCM group compared to healthy controls. The authors did not find reasonable explanations for the elevated levels of IL-6 in HCM patients.

Although it is known that inflammation contributes to CHF progression, no effective anti-inflammatory therapies capable of reducing CHF progression have been yet developed [31, 32]. So far, two large randomized clinical trials evaluated the use of cytokine inhibition in CHF therapy targeting TNF-alpha as the most extensively explored cytokine in CHF: the RENEWAL trial used etanercept: a recombinant human TNF receptor that binds to circulating TNF and functionally inactivates it; in the ATTACH trial infliximab was used, a monoclonal antibody against TNF-alpha. Although inhibition of TNF-alpha is a proven therapeutic concept in patients suffering from rheumatoid arthritis and psoriasis, no clinical benefits could be observed using anti-inflammatory drugs in patients with CHF yet. The observation that ivabradine leads to a significant increase in the number of myeloid DCs almost to numbers of healthy individuals might be a first step in focusing on anti-inflammatory properties of medical treatment in CHF.

Despite all the improvements observed via ivabradine therapy, certain limitations of our present study have to be mentioned. (1) The number of patients recruited into our study and in particular in the subgroups were relatively small which reduces statistical power and might be responsible for the fact that we often got borderline statistical significant results. (2) The duration of the ivabradine therapy evaluated in this study was only 6 months, which is a relatively short time; hence long-term changes such as remodeling could not be evaluated. This might also explain the fact that, in echocardiography, we only observed significant changes in EF, but not, for instance, changes in LVEDd. (3) An uncommonly large proportion of patients (*n* = 17) dropped out of the study due to rather unspecific side effects. We assume that this might be associated with negative expectations of the patients about possible side effects of a medication evaluated in a clinical trial. (4) The comparison of the different dosages of ivabradine is limited, since the dosage could be elevated only in two-thirds of the study patients after 3 months. (5) The study was not double-blinded; therefore a suggestive effect, particularly regarding the improvement of CHF symptoms, cannot be excluded.

The present study demonstrates that ivabradine exerts certain immunomodulatory properties that might contribute to its global beneficial effect in CHF. Whether these effects result from heart rate reduction or represent a heart rate independent mechanism needs to be further investigated. The RIVERA study is one pilot study which will analyze anti-inflammatory effects of ivabradine in more detail [33]. However, this study is designed for a population of patients with acute coronary syndrome. Similar studies are necessary to evaluate anti-inflammatory effects of ivabradine in patients with CHF.

## 5. Conclusions

In the present study, we show an improvement of CHF symptoms, echocardiographic and serological CHF parameters, and pro-inflammatory mediators during ivabradine treatment in CHF. Immunological changes induced by ivabradine are more pronounced in patients with dilated or ischemic cardiomyopathy compared to hypertensive cardiomyopathy. It needs to be further elucidated whether these effects are a result of heart rate reduction, or possibly an additional (pleiotropic) effect of ivabradine. However, the fact that the heart rate reduction was very similar in DCM, ICM, and HCM, whereas significant immunological changes were only observed in DCM and ICM, might be the first hint to a heart rate independent effect of ivabradine. Further studies are needed to evaluate this issue.

## Figures and Tables

**Table 1:** Baseline characteristics of the study participants.

	DCM (*n* = 11) mean ± SEM OR *n* (%)	ICM (*n* = 12) mean ± SEM OR *n* (%)	HCM (*n* = 10) mean ± SEM OR *n* (%)	*p* value
Age [in years]	55 ± 12	60 ± 8	62 ± 14	*n.s.*
Male	9 (81)	11 (92)	7 (70)	*n.s.*
BMI [in kg/m^2^]	31 ± 2.9	29 ± 5.7	33 ± 5.6	*n.s.*
*Cardiovascular risk factors*				
Hypertension	6 (54)	9 (75)	11 (100)	*<0.001*
Diabetes mellitus	5 (45)	8 (66)	4 (40)	*n.s.*
Smoking	3 (27)	8 (66)	1 (10)	*n.s.*
Dyslipidaemia	5 (45)	8 (66)	6 (60)	*n.s.*
Obesity (BMI > 30 kg/m^2^)	8 (73)	7 (58)	6 (60)	*n.s.*
*Medication*				
Beta blockers	11 (100)	12 (100)	10 (100)	*n.s.*
ACE-inhibitors/ARB	11 (100)	12 (100)	10 (100)	*n.s.*
Aldosterone antagonists	7 (64)	8 (66)	6 (60)	*n.s.*
*Clinical Presentation*				
Angina pectoris	0 (0)	1 (8)	1 (10)	*n.s.*
NYHA class	2.2 ± 0.6	2.2 ± 0.9	2.4 ± 1.0	*n.s.*
Peripheral oedema	1 (9)	3 (25)	3 (30)	*n.s.*
Heart rate	80 ± 8.1	80 ± 4.8	80 ± 7.4	*n.s.*
RR sys	123 ± 16	148 ± 21	149 ± 26	*n.s.*
RR dia	82 ± 12	88 ± 11	87 ± 11	*n.s.*
Floors	2.4 ± 1.2	2.3 ± 1.1	2.4 ± 1.9	*n.s.*
*Echocardiography*				
LV-EF [in %]	31 ± 8	35 ± 8	33 ± 12	*n.s.*
LVEDd [in mm]	61 ± 8	59 ± 11	66 ± 11	*n.s.*
PAPsys [in mmHg] (excl. CVP)	24 ± 6	19 ± 9	21 ± 2	*n.s.*

ARB: aldosterone-receptor blocker, BMI: body mass index, CVP: central venous pressure, DCM: dilated cardiomyopathy, HCM: hypertensive cardiomyopathy, ICM: ischemic cardiomyopathy, LVEDd: left ventricular end-diastolic diameter, LV-EF: left ventricular ejection fraction, NYHA: New York Heart Association, and PAPsys: systolic pulmonary artery pressure.

**Table 2:** Answers to the SF-36 questionnaire of the study participants. Data presented as mean ± SEM for the baseline investigation, 3-month follow-up, and 6-month-follow-up. Follow-up data were compared to the baseline measurements and statistical significance considered as a *p* value < 0.05 is presented in comparison to the baseline answer.

	Baseline	3-month follow-up		6-month follow-up	
	Mean ± SEM	Mean ± SEM	*p value*	Mean ± SEM	*p* value
*GENERAL HEALTH (1-excellent, 2-very good, 3-good, 4-fair, 5-poor)*					
How is your health in general	3.24 ± 0.09	3.06 ± 0.12	*NS*	2.96 ± 0.12	*p* = 0.04

*PHYSICAL HEALTH PROBLEMS during the past 4 weeks that resulted in problems with work or other regular daily activities (1-yes, 2-no)*					
Cut down the amount of time you spent on work or other activities	1.48 ± 0.07	1.18 ± 0.08	*p* = 0.003	1.96 ± 0.04	*p* < 0.001
Were limited in the kind of work or other activities	1.56 ± 0.07	1.82 ± 0.08	*p* = 0.02	1.96 ± 0.04	*p* < 0.001
Had difficulty performing the work or other activities	1.50 ± 0.07	1.76 ± 0.09	*p* = 0.03	1.06 ± 0.04	*p* < 0.001

*EMOTIONAL HEALTH PROBLEMS during the past 4 weeks that resulted in problems with work or other regular daily activities (1-yes, 2-no)*					
Cut down the amount of time you spent on work or other activities	1.65 ± 0.07	1.88 ± 0.07	*p* = 0.03	1.96 ± 0.04	*p* = 0.002
Accomplished less than you would like	1.65 ± 0.07	1.88 ± 0.06	*p* = 0.02	1.92 ± 0.05	*p* = 0.009
Didn't do work or other activities as carefully as usual	1.73 ± 0.06	1.94 ± 0.04	*p* = 0.016	1.96 ± 0.04	*p* = 0.014

*SOCIAL ACTIVITIES (1-not at all, 2-slightly, 3-moderately, 4-severe, 5-very severe)*					
Emotional problems interfered with your normal social activities	1.67 ± 0.14	1.36 ± 0.13	*NS*	1.08 ± 0.08	*p* = 0.006

NS = not significant.

## Abbreviations

ACE: Angiotensin converting enzyme
APC: Antigen-presenting cell
ARB: Angiotensin receptor blocker
Bpm: Beats per minute
CAD: Coronary artery disease
CD: Cluster of differentiation
CVP: Central venous pressure
DC: Dendritic cell
DCM: Dilated cardiomyopathy
DCP: Dendritic cell precursor
HCM: Hypertensive cardiomyopathy
HR: Heart rate
ICM: Ischemic cardiomyopathy
IL: Interleukin
LV: Left ventricular
LVEDd: Left ventricular end-diastolic diameter
LV-EF: Left ventricular ejection fraction
NYHA: New York Heart Association
PAPsys: Systolic pulmonary artery pressure
RR: Blood pressure.
